# miRNAs in Heart Development and Disease

**DOI:** 10.3390/ijms25031673

**Published:** 2024-01-30

**Authors:** Estefania Lozano-Velasco, José Manuel Inácio, Inês Sousa, Ana Rita Guimarães, Diego Franco, Gabriela Moura, José António Belo

**Affiliations:** 1 Cardiovascular Development Group, Department of Experimental Biology, University of Jaen, 23071 Jaen, Spain; evelasco@ujaen.es (E.L.-V.); dfranco@ujaen.es (D.F.); 2 Stem Cells and Development Laboratory, iNOVA4Health, NOVA Medical School|Faculdade de Ciências Médicas, Universidade NOVA de Lisboa, 1150-082 Lisbon, Portugal; jose.inacio@nms.unl.pt; 3 Genome Medicine Lab, Department of Medical Sciences, Institute for Biomedicine–iBiMED, University of Aveiro, 3810-193 Aveiro, Portugal; inesmmsousa@ua.pt (I.S.); rita.guimaraes@ua.pt (A.R.G.); gmoura@ua.pt (G.M.)

**Keywords:** miRNA, heart development, cardiovascular diseases, bioinformatic tools

## Abstract

Cardiovascular diseases (CVD) are a group of disorders that affect the heart and blood vessels. They include conditions such as myocardial infarction, coronary artery disease, heart failure, arrhythmia, and congenital heart defects. CVDs are the leading cause of death worldwide. Therefore, new medical interventions that aim to prevent, treat, or manage CVDs are of prime importance. MicroRNAs (miRNAs) are small non-coding RNAs that regulate gene expression at the posttranscriptional level and play important roles in various biological processes, including cardiac development, function, and disease. Moreover, miRNAs can also act as biomarkers and therapeutic targets. In order to identify and characterize miRNAs and their target genes, scientists take advantage of computational tools such as bioinformatic algorithms, which can also assist in analyzing miRNA expression profiles, functions, and interactions in different cardiac conditions. Indeed, the combination of miRNA research and bioinformatic algorithms has opened new avenues for understanding and treating CVDs. In this review, we summarize the current knowledge on the roles of miRNAs in cardiac development and CVDs, discuss the challenges and opportunities, and provide some examples of recent bioinformatics for miRNA research in cardiovascular biology and medicine.

## 1. Introduction

### 1.1. Non-Coding RNA Biogenesis, Function, and Molecular Evolution

Non-coding RNAs can be classified according to their length into two broad categories: small non-coding RNAs that are <200 nt in length and long non-coding RNAs that are >200 nt in length [1,2]. Among small non-coding RNAs, microRNAs constitute the most well-studied subgroup [3,4]. Mature microRNAs are 22–24 nt in length and are encoded from precursor microRNA genes, named pre-miRNAs, with an approximate length of 120–160 nt. In certain cases, microRNA genes can be clustered in the genome and thus are transcribed into a single precursor molecule termed pri-miRNA [3,4]. The biogenesis of microRNAs has been studied in detail over recent decades. Most microRNAs are transcribed by polymerase II, exported to the cytoplasm by exportin 5 complex, matured by endonuclease Dicer, and subsequently loaded into the RISC complex, in which one of the passenger strands are degraded [3,4,5]. The remaining strand scans for target molecules by base pair complementarity and evokes RNA instability and/or translational blockage [3,4,5]. microRNAs can also be silently kidnapped by other non-coding RNA molecules, such as circRNAs and/or lncRNAs [6,7]. Importantly, microRNAs are very well conserved among evolution, ranging from C. elegans to humans [8], in contrast to other non-coding RNAs, such as lncRNAs and circRNAs [9,10].

### 1.2. Bioinformatic Tools of Non-Coding RNAs

Non-coding RNAs (ncRNAs) have emerged as key regulators in the pathogenesis and progression of heart failure (HF). However, understanding the intricate regulatory roles of ncRNAs demands increasingly sophisticated bioinformatics methodologies to elucidate their expression, interactions, and functional implications. As an example of this trend, more than 1000 bioinformatics tools have been developed for miRNA study since 2003, of which those dedicated to identification and target prediction take the lead [11]. Table 1 summarizes some of the bioinformatics tools that have been used to uncover the main ncRNAs present in RNA sequencing data and their features.

### 1.3. An Introduction to Cardiac Development and Disease

Cardiogenesis is a complex developmental process involving the contribution of distinct cell types [34,35]. Cardiogenic precursors are specified soon after gastrulation as two bilateral mesodermal cell subpopulations. Soon thereafter, the cardiogenic precursors merge in the embryonic midline, leading to the formation of a cardiac straight tube that is uniquely composed of an inner endocardial and an outer myocardial layer [34,35]. As development proceeds, the cardiac tube invariably displays a rightward displacement, configuring the first sign of embryonic asymmetry and leading to the formation of the primitive arterial and venous poles of the heart. Subsequently, atrial and ventricular chambers are formed, and the embryonic heart is externally lined by the third cardiac layer, the epicardium. The atrial and ventricular chambers progressively mature with the formation of compact and trabeculated components in the ventricular chambers and pectinated muscle in the atrial chambers [34,35]. Additionally, endocardial cushions are formed at the atrioventricular and conotruncal regions, configuring the primitive cardiac valves. Finally, all the embryonic cardiac components are divided by a complex process of septation, leading to the formation of a four-chambered organ with separated inlet and outlet connections [34,35], a process that is partly directed by the contribution of cardiac neural crest cells.

As a final result of these coordinated embryonic developmental processes, the adult heart is configured [36]. The adult heart is composed of three layers, the endocardium, myocardium, and epicardium, and is irrigated by a complex coronary vasculature with distinct left and right coronary arteries [37]. A specialized cardiac conduction system with slow conducting nodes, the sinoatrial and the atrioventricular nodes, respectively, and fast conducting branches, such as the common branch of the bundle of His and the left and right bundle branches, is also configured [38,39,40]. Furthermore, the aortic and semilunar valves provide a secure blood flow closure at the ventricular chambers, while the atrioventricular valves, i.e., the tricuspid and mitral valves, act similarly at the atrioventricular junctions [34,35].

The impairment of any of the previously described developmental processes caused by genetic and/or environmental factors invariably leads to congenital cardiac malformations [41,42,43]. In fact, congenital heart diseases are the most prevalent type of congenital defects in the human population [44]. The most frequent alterations occur as a consequence of abnormal septation, i.e., atrial septal defects, ventricular septal defects, or atrioventricular septal defects. In more rare cases, complex cardiac abnormalities occur, such as those primarily affecting left-sided obstructions, i.e., hypoplastic left heart syndrome (HLHS) and aortic stenosis, or those encompassing cyanotic heart diseases such as tetralogy of Fallot or Epstein’s anomaly.

Similarly, adult cardiovascular diseases are the leading cause of death worldwide according to the World Health Organization. Cardiovascular alterations can be broadly classified between those affecting the coronary vasculature, leading, therefore, to impaired cardiac perfusion and thus to ischemic heart disease [45]; those affecting the electrical wiring of the heart, such as, e.g., cardiac conduction defects, and atrial and ventricular fibrillation; those affecting the structure of the myocardial walls, such as dilated and hypertrophic cardiomyopathy; and those affecting the cardiac valves, i.e., valvulopathies [46,47,48,49].

In the next paragraphs, a state-of-the-art review of the contribution of microRNAs to cardiac development and diseases is provided, including their role in both cardiogenesis as well as in distinct congenital and adult cardiac diseases.

## 2. The Role of microRNAs in Heart Development

Our current understanding of the functional role of microRNAs during human heart development is scarce. Evidence of their role is only available in surrogated experimental models, such as human embryonic stem cells and/or human induced pluripotent stem cell differentiation into cardiomyocytes [50,51,52,53,54,55,56], highlighting the role of miR-1 [56], miR-499 [56], miR-10 [50], miR-363 [52], miR-200c [53], and let7 [54] during cardiomyocyte maturation. On the other hand, seminal work in murine experimental models by Zhao et al. (2007) demonstrated the critical role of microRNAs during cardiac development since the deletion of Dicer, a key microRNA endonuclease-processing enzyme in Nkx2.5-expressing cells, led to embryonic cardiac lethality [57]. Since then, a large body of evidence has reported the fundamental role of distinct microRNAs during all stages of cardiac development using different in vitro and in vivo experimental models, as recently reviewed by Lozano-Velasco et al. (2022) [58]. For example, miR-130 and miR-133 play fundamental roles in orchestrating cardiomyogenic specification by Fgf8 and Bmp2 during precardiac mesoderm formation in chicken embryos [59,60], while miR-99a/let-7c are involved in the control of cardiomyogenesis, in part, by altering epigenetic factors, as reported in mouse embryonic stem cells [61]. Similarly, the deployment of the heart field and the establishment of cardiac sidedness and looping are also influenced by microRNAs. Key transcription factors regulating first and second heart field formation, such as Tbx5 and Isl1, are modulated by distinct microRNAs, such as miR-17 (Isl1), miR-20 (Isl1), and miR-200 (Tbx5) [62,63]. Similarly, the establishment of cardiac sidedness and particularly those processes regulated by the Prrx1 transcription factor are modulated by microRNAs such as miR-34a and miR-92a [64]. The formation of the epicardium and its derivatives is also modulated by microRNAs. The seminal work by Singh et al. (2011) demonstrated that deletion of Dicer in Gata5-Cre expression domains led to impaired epicardium development and thus thin ventricular myocardium and coronary vasculature anomalies [65]. Since then, the role of discrete microRNAs in epicardial development has been reported, such as miR-21 [66], miR-200c [67] promoting the epithelial-to mesenchymal transition, and miR-195 and miR-223 promoting cardiomyogenic differentiation [68]. Finally, the post-transcriptional control of microRNAs during cardiac conduction system and chamber morphogenesis has also been reported, particularly modulating the expression of Tbx2 and Tbx3 transcription factors in cardiac conduction development and Hand1 and Hand2 during chamber morphogenesis, among others (see for a detailed review [58]). In sum, a large body of evidence has reported on the crucial role of microRNAs in different stages of cardiac development using different experimental models, supporting the notion that such modulation will be similarly important during human cardiogenesis.

## 3. The Role of microRNAs in Congenital Heart Diseases

As previously mentioned, congenital heart diseases are the most frequent type of congenital anomalies in humans [44]. Among them, the most prevalent type of cardiac congenital anomaly is caused by impaired atrial (ASD), ventricular (VSD), or atrioventricular (AVSD) septation. Currently, the involvement of microRNAs in these cardiac pathologies in humans has only been described for VSD. Different microRNA polymorphisms, such as those in miR-196, miR-27, and miR-499, have been associated with distinct CHDs, particularly septal defects [69]. Furthermore, several studies reported distinct microRNAs as biomarkers of ASD [70], VSD [71], and AVSD [72]. Importantly, Li et al. (2014) detected 36 microRNAs that dysregulated expression in the circulating plasma of VSD patients [73]. Seven of these microRNAs, namely, let-7e, miR-155, miR-222, miR-379, miR-409, miR-433, and miR-487, were down-regulated, while miR-498 was up-regulated. While emerging evidence of the dysregulation of different microRNAs has been reported in human VSD, mechanistic insights into their contribution are still scarce.

Additional evidence of the plausible role of microRNAs in ventricular septal defects has been obtained using mouse experimental models, as recently reviewed by Dueñas et al. (2019) [74]. miR-1-2-deficient mice resulted in heart defects that include VSDs [57,75]. Interestingly, the single deletion of miR-133a-1 and miR-133a-2 microRNAs in mice does not result in any cardiac pathology; however, the combined deletion of both genes led to late embryonic and neonatal deaths due to VSD and cardiac chamber dilatation. Finally, targeted deletion of the miR17-92 family of microRNAs results in neonatal lethality due to lung hypoplasia and VSDs [76].

A second group of cardiac congenital heart diseases is represented by left-sided obstructive lesions, such as hypoplastic left heart syndrome (HLHS), aortic stenosis, aortic coarctation, mitral stenosis, and interrupted aortic arches (IAA). To date, microRNAs have only been reported in the context of HLHS. Sucharov et al. (2015) reported the microRNA profile of the RV in HLHS patients compared with the RV of non-failing hearts and identified a total of 93 microRNAs differentially regulated in HLHS hearts [77]. Furthermore, such a microRNA profile displays scarce similarities as compared to pediatric and adult idiopathic dilated cardiomyopathy, suggesting an microRNA-specific hallmark for HLHS.

Finally, a third group of cardiac congenital heart diseases encompasses cyanotic heart diseases, such as transposition of the great arteries (TGA), tetralogy of Fallot, Ebstein´s anomaly, double-outlet right ventricle (DORV), permanent truncus arteriosus (PTA), and total anomalous pulmonary venous connection. At present, the role of microRNAs has only been described in TGA, TOF, and DORV, as recently reviewed by Dueñas et al. (2019) [74].

Seminal studies reported that distinct microRNAs might serve as biomarkers of adult TGA [78], although such differences were not replicated in another independent study [79]. Therefore, additional studies are required to clarify this controversy.

Several studies have provided evidence of genetic associations between single-nucleotide variants in distinct microRNAs and the occurrence of TOF, particularly on miR-17 [80] and miR-196 [81], yet their functional implication is still unclear. Furthermore, evidence on the involvement of multiple deregulated microRNAs in distinct conditions of TOF, including pediatric [82,83,84,85,86,87] and corrected adult patients, have been reported [88,89]. For example, O’Brien et al. (2012) identified 61 microRNAs that display significant changes in expression levels in children with TOF [83]. Interestingly, these microRNA levels remain similar to those in the normal fetal myocardium. Furthermore, these authors identified 33 microRNAs that were significantly downregulated in TOF myocardial tissue compared to the normal myocardium. Zhang et al. (2013) also described 18 differentially expressed microRNAs in TOF patients, demonstrating the role of miR-424 and miR-222 in regulating cardiomyocyte proliferation [84]. In addition, analyses of microRNA and mRNA complementary expression identified the functional relationship between miR-421 and SOX4 expression in TOF patients, as described by Bittel et al. (2014) [85]. In sum, these data exemplify the emerging role of microRNAs in complex congenital heart defects, such as TOF.

While there are no reports of the implication of microRNAs in DORV in humans, experimental evidence in mice unraveled the fundamental role of microRNAs in DORV development. Conditional deletion of Dicer in either the developing embryonic heart [90] or specifically in the cardiac neural crest cells [91] leads to DORV. In sum, these data demonstrate the emerging role of distinct microRNAs in different cardiac congenital heart diseases.

## 4. The Role of microRNAs in Atrial Fibrillation

Atrial fibrillation (AF) is the most frequent type of cardiac arrhythmia in humans, with an estimated incidence of 1–2% in the general population, rising up to 8–10% in the elderly. AF is characterized by irregular electrical beats in the atrial chambers. Such abnormal electrical patterning, if sustained, leads to both electrical and structural remodeling, modifications that are directly linked to the course of AF. Electrical remodeling is characterized by disturbed expression of the cardiac action potential and cell–cell intercellular components, while structural remodeling primarily involves atrial dilation that is frequently accompanied by apoptosis, atrial fibrosis, and/or inflammation.

Multiple studies have reported the implication of different microRNAs in regulating distinct phases of the cardiac action potential by modulating the expression of potassium, sodium, calcium, and cation channels, as reviewed by Lozano-Velasco et al. (2020) and Franco et al. (2020) [92,93]. Among the most well-studied and characterized microRNAs, miR-1 targets different ion channels involved in the human resting membrane potential (KCNJ2, KCNE1, KCNB2) [94] and nodal-like upstroke current (HCN2, HCN4) [95], while additional evidence is also supported in experimental models [96]. Other microRNAs, such as miR-26, miR-30d, miR-499, miR-192, miR-21, miR-29, miR-208, miR-328, miR-106b-25, and miR-206 have also been reported to contribute to the electrical remodeling in AF (see for a recent review [92]).

Structural remodeling in AF, particularly atrial fibrosis, is modulated by multiple microRNAs. Yang et al. (2019) reported that miR-23b and miR-27b overexpression enhances the up-regulation of fibrosis-associated genes by targeting transforming growth factor beta receptor 3 (TGFBR3) and posterior activation of SMAD3 signaling in human cardiac fibroblasts [97]. miR-26 is down-regulated in AF in humans as well as in different experimental models of AF, providing the cues for increasing TRPC3 expression, which, in turn, stimulates fibroblast proliferation, differentiation, and activation [98]. miR-29 targets multiple extracellular matrix genes, including collagens, fibrillins, and elastin. miR-29 is downregulated in human AF patients as well as in AF experimental models. Its expression is inversely correlated with extracellular matrix protein levels and the development of AF [99].

Additional evidence is also supported by different experimental models. For example, miR-21 represses sprouty RTK signaling antagonist 1 (SPRY1) [100] and promotes cardiac fibrosis through the transcription factor signal transducer and activator of transcription 3 (STAT3) signaling pathway [101] in rats. Furthermore, miR-30a up-regulation reduces AF-induced myocardial fibrosis by targeting snail family transcriptional repressor 1 (SNAIL1) in a rabbit model of experimental AF [102], whereas miR-30c overexpression attenuates Tgf-β1-induced atrial fibrosis by targeting transforming growth factor beta receptor 2 (TgfβrII) [103].

Another component of AF structural remodeling is apoptotic cell death. In this context, miR-133 has a cardioprotective role in homeostasis but induces apoptosis in AF human patients if down-regulated [104]. Similarly, it has been demonstrated that miR-122 is up-regulated in AF human patients, while in a mouse AF experimental model, it inhibits ERK activation and thus leads to apoptosis [105]. Additionally, Fu et al. (2021) demonstrated that miR-520d suppresses rapid pacing-induced apoptosis of atrial myocytes through the mediation of ADAM10, and Yu et al. (2019) reported that miR-23 suppresses fibroblast apoptosis in AF through targeting Tgf-β1 [106,107]. Overall, these studies illustrate the relevant role of different microRNAs in AF structural remodeling.

## 5. The Role of miRNAs in Heart Failure and Fibrosis

Heart failure (HF) is a condition in which the heart cannot pump enough blood to meet the organism’s demand. Heart failure with preserved ejection fraction (HFpEF) and heart failure with reduced ejection fraction (HFrEF) are the two main pathophysiological manifestations of heart failure (HF), with a five-year mortality rate of 75% [108]. HFpEF is characterized as HF with preserved left ventricular ejection fraction (LVEF ≥ 50%) by impaired ventricular relaxation, increased diastolic stiffness, and fibrosis of the myocardium as a result of a systemic inflammation due to comorbidities such as hypertension, diabetes, hyperlipidemia, obesity, chronic kidney disease, and others [108,109]. HFrEF is characterized by a structural and functional impairment of the left ventricle, resulting in a decrease in heart pump function (LVEF ≤ 40%), which is associated with impaired exercise tolerance, dyspnea, edema, fatigue, and others [110]. HFmrEF, heart failure with mid-range ejection fraction, represents an HF group of patients with heterogeneous clinical characteristics that fall between HFpEF and HFrEF with both mild systolic and mild diastolic dysfunction and LVEF between 40% and 50% [111].

Though several microRNAs have been identified and associated with HF of different etiologies (see Table 2), the molecular relevance of these microRNAs in the HF pathophysiological process continues to be elusive, in part due to its associated comorbidities. On the other hand, miR-21 is one of the few well-studied microRNAs implicated in the development of HFpEF and pathogenesis of myocardial fibrosis [112]. It has been experimentally shown that miR-21 can modulate cardiac remodeling and dysfunction by affecting the proliferation, hypertrophy, apoptosis, and fibrosis of different cell types in the heart [112]. Increased miR-21 levels associated with increased levels of TGF-β activated the transcription of pro-fibrotic factors via the SMAD2 and SMAD7 pathway [113]. Moreover, in a PTEN/AKT-pathway-dependent manner, it was described that miR-21-TGF-β signaling stimulates endothelial-to-mesenchymal transition, increasing the number of cardiac fibroblasts and the availability of fibrogenic cells during cardiac remodulation [114]. Also associated with EndoMT, Cheng et al. showed that *programmed cell death 4* (*PDCD4*) was downregulated by miR-21, thus resulting in less cardiac cell death and apoptosis [115]. MiR-21 was also found to suppress the apoptosis of cardiac fibrogenic cells by stimulating another antiapoptotic gene, *BCL-2*, thus promoting the development of HFpEF [116]. Using ventricular myocardial biopsies from patients with HFpEF, a recent study led to the identification of new miRNA–mRNA relationships in HFpEF by bioinformatic analysis [117]. Moreover, the authors further experimentally tested the predicted miRNA–mRNA interactions using a cardiomyocyte primary cell line and validated them by real-time PCR. In this way, it was demonstrated that the upregulation of miR-25, miR-26a, and miR4429 led to the downregulation of *hyaluronic acid (HA)-organizing factor*, *HAPLN1*, which is required for the production of the hyaluronic-rich matrix during heart morphogenesis and injury-induced remodeling [117]. Also confirmed was the interaction of miR-26a and miR-140 in the regulation of *NPPB*, a cardiac gene encoding brain natriuretic peptide (BNP), which is strongly expressed in ventricular cardiomyocytes when the heart is overloaded [117].

Moreover, miRNAs could also be used as biomarkers for the differentiation of HF phenotypes, particularly the circulating miRNAs [139]. For example, HFpEF and HFrEF present similar clinical manifestations, but mortality is somewhat lower in HFpEF than in HFrEF. Curiously, HFpEF patients are less responsive to the available therapeutic approaches compared to HfrEF. Therefore, profiling miRNA in HF subtyping should be considered a potential tool for diagnostics and prognostics to differentiate HFrEF from HFpEF. Indeed, the diagnostic performance of HF was improved when the levels of circulating miRNA were part of a differential diagnostic panel [139]. In this study, five miRNAs were found to be reduced in HF (miR-30c, miR-146a, miR-221, miR-328, and miR-375), with miR-375 only reduced in HFrEF. In addition, Inácio and colleagues also observed that miRNA signatures could be further used to distinguish HFpEF sub-groups [117].

Therefore, these pieces of evidence emphasize the role of microRNAs in the different HF pathophysiological processes, as well as in their ability to meet the need for accurate and faster HF differential diagnosis.

## 6. The Role of miRNAs in Coronary Artery Disease

Coronary artery disease (CAD) is a condition that affects the blood supply to the heart muscle by the coronary arteries [140]. The leading cause of CAD is atherosclerosis, a chronic inflammation of the wall of the arteries causing a malfunction in the vascular endothelial cells, which become narrowed or blocked by plaque [141]. Distinct studies have shown that miRNAs are involved in the pathogenesis and progression of CAD, modulating the expression of key genes involved in molecular processes such as inflammation, lipid metabolism, oxidative stress, apoptosis, angiogenesis, and others [142,143,144,145]. miR-126 is one of the key miRNAs in CAD and is consistently downregulated in the disease context. This miRNA directly inhibits the expression of the *vascular adhesion molecule 1* (*VCAM-1*) and impacts tumor necrosis factor-alpha (TNF-α), resulting in an anti-inflammation effect [145]. Moreover, miR-126 is implicated in the regulation of the vascular endothelial factor (VEGF) pathway and in inhibiting SPRED1 and PIK3R2, which are two inhibitors of mitogen-activated protein kinase (MAPK) and phosphatidylinositol kinase (P13k), respectively [143]. Another relevant microRNA involved in the development and progression of CAD is miR-21 [146]. As observed above, the upregulation of miR-21 promotes cardiac fibrosis, a CAD complication [142]. CAD also involves the upregulation of miR-155 [147]. It has been demonstrated that the inhibition of this microRNA promotes pro-resolving atherosclerotic plaque microenvironments, decreasing the amount of pro-inflammatory cytokines and promoting a shift from M1 to M2 macrophages, which promotes cell proliferation and repair [147]. In sum, these studies demonstrated the involvement of miRNAs in the etiology of CAD and therapeutic targets.

## 7. The Role of miRNAs in Myocardial Infarction

Myocardial infarction is a life-threatening condition that occurs when one or more of the coronary arteries that supply blood to a part of the heart muscle is blocked [148]. When the blood flow to the heart is interrupted, the myocardial tissue can suffer irreversible damage or death [149]. This is characterized by structural alterations involving heart chamber dilation and ventricular wall thinning caused by cardiomyocyte apoptosis, an increase in extracellular matrix, fibrosis, and the hypertrophy of cardiac myocytes, which produce more clinical complications leading to heart failure [149].

Recent studies have shown that miR-1, miR-133 and miR-499 are cardiac-specific miRNAs released into the circulation after MI and reflect the extent of myocardial injury. For example, miR-1 in serum is also positively associated with myocardial infarct size [150]. Moreover, miR-1 is involved in AMPK pathway regulation, promoting cardiomyocyte apoptosis [151]. A miRNA frequently associated with MI is miR-133 [152]. By targeting multiple genes and signaling pathways, such as RhoA, MAPK, TGFβ/Smad, and PI3K/Akt, miR-133 modulates the survival, hypertrophic growth, and electrical conduction of cardiomyocytes [153]. miR-499 is a microRNA expressed in the heart, regulating the expression of several genes involved in cardiac contractility, calcium handling, and energy metabolism [154]. Overexpression of miR-499 alters the expression of contractile activity (*MYH7B*) and skeletal muscle α-actin (*ACTA1*) and conductivity (*KCNH6* and *Kv11.1*), which leads to heart hypertrophy and cardiac impairments [155].

miR-21, miR-146a, and miR-155 are also dysregulated after MI and are associated with adverse outcomes by their involvement in the inflammation and immune responses, as already discussed above [156]. Curiously, miR-126, miR-210, and miR-378 are involved in angiogenesis and myocardial repair after AMI and may have protective effects [157]. For example, miR-126 directly *represses sprout-related EVH1 domain-containing protein 1* (*SPRED1*), *vascular cell adhesion molecule 1* (*VCAM1*), and *phosphatidylinositol 3-kinase* (*PIK3*) *regulatory subunit beta R2/p85-beta* (*R2/p85-beta*), which negatively regulate vascular endothelial growth factor (VEGF) signaling [158]. These results suggest that the modulation of specific miRNAs may have therapeutic potential by impacting the different cells’ overall survival and regenerative capacity in an injured heart.

## 8. Novel Bioinformatic Tools for the Study of ncRNAs

Historically, bioinformatics tools for ncRNA research started to be developed for specific purposes, mainly identification, curation and target prediction. Many of these tools however (see Table 1 for a not systematic list), rely on bioinformatics algorithms that filter results based on a stringent seed-based match, yielding a high false-positive rate and a reduced overlap among them [159]. Consequently, we assisted in the identification of tools capable of crossing expression profiles (miRNA vs. mRNA) to detect more solid functional associations, while other criteria such as gene ontology and pathway analysis started being incorporated into the decision process. More recently, bioinformatics integrated tools have emerged (e.g., miRDeep2 [13], Tools4miRs [160]), and new knowledge about ncRNA biology is starting to give rise to a new burst of dedicated tools, such as those to study isoforms of miRNAs (isomiRs) or their chemical modifications. In the meanwhile, many early tools are still being widely used and maintained [11], being freely available to the scientific community. Interestingly, in the last 10 years, these tools have shifted their main focus towards the study of disease-associated ncRNAs.

**Figure 1:**
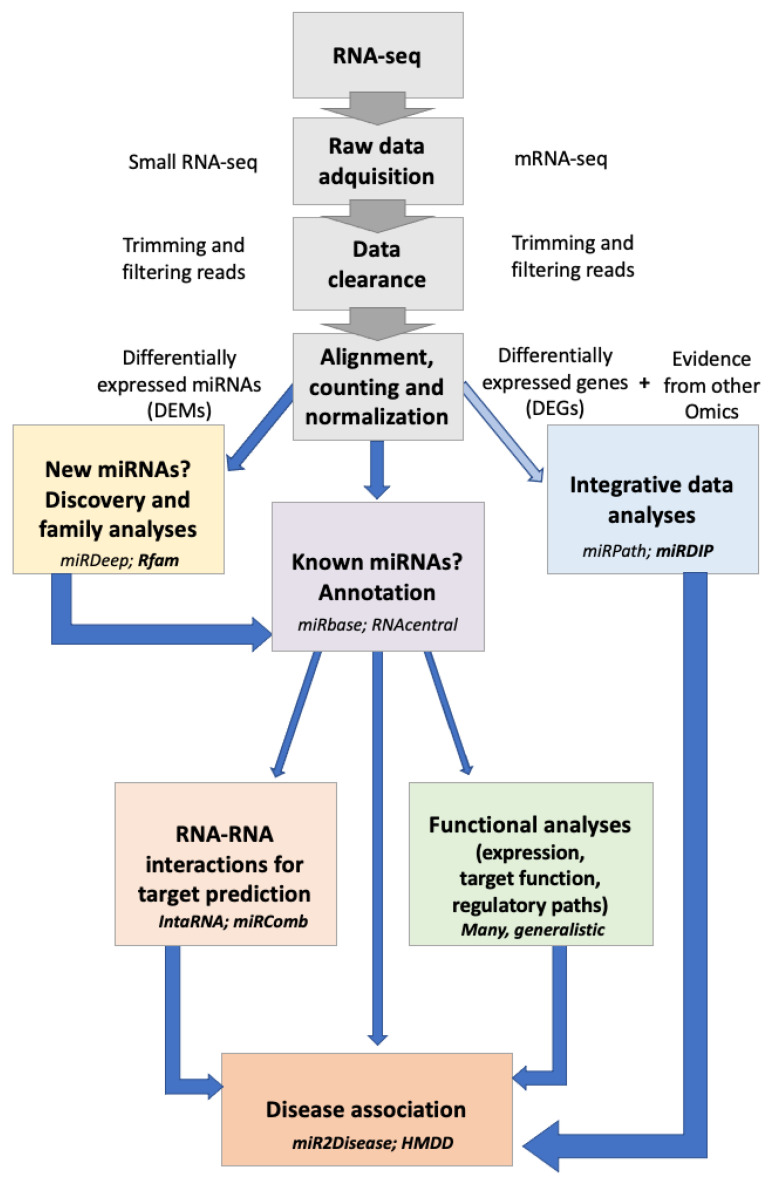
Proposed workflow for miRNA bioinformatic analysis. The process commences, depicted in grey, with the RNA pipeline designed for conducting differential expression analysis (for miRNA, mRNA, and eventually other omics of interest). Subsequently, the workflow encompasses miRNA discovery and annotation steps, as well as downstream cause-and-effect analyses. Each uniquely colored box corresponds to a distinct category of advanced bioinformatics tools, as elaborated in the main text and Table 3. Within each box, common tools are exemplified in italics, while novel ones are highlighted in bold.

Next, categorized by their primary functionalities as explained above, we highlight a set of novel bioinformatics tools used in studying ncRNAs (see also Table 3), particularly in the context of cardiovascular research (Figure 1).

### 8.1. Bioinformatics Tools for ncRNA Family Identification

Several databases gather miRNA sequences from different species so that conserved structures or targets can be discovered, giving a rapid overview of the genomic location of miRNAs or other regulatory RNAs. These include the database Rfam, which contains curated families of ncRNA sequences and alignments, allowing for the annotation of non-coding RNA genes in genomic sequences [161]. Complementarily to Rfam, there is Infernal, a software suite for searching DNA sequence databases for homologs of ncRNA families using covariance models (CMs) [162]. It is particularly useful for identifying remote homologs of structured RNA.

Since they both assist in identifying conserved RNA families, Infernal and Rfam have aided in the identification and annotation of novel non-coding RNA genes, as well as in understanding their evolutionary conservation. As a possible approach, gene families represented in Rfam can be extracted (e.g., in Stockholm or flat file format) and aligned using Infernal, giving insight about the roles of ncRNAs, as consistently annotated sets of orthologous and paralogous ncRNA genes become available [183].

### 8.2. Bioinformatics Tools for RNA–RNA Interaction Prediction

Presently, there are many bioinformatic algorithms for miRNA target prediction that are based on sequence and location characteristics. However, they differ on the weight given to each different parameter, e.g., seed match, phylogenetic conservation, free energy, etc. [159].

One such tool is IntaRNA, whose algorithm considers both sequence complementarity and target accessibility to predict RNA–RNA interactions, and hence, it has been used to pinpoint the interplay between ncRNAs and their target RNAs, not necessarily mRNAs. In order to achieve this, the user needs to provide the two RNA sequences (minimum) whose interaction is to be analyzed, usually in plain text, FASTA, or other standard-sequence format. IntaRNA [163] can be employed in heart failure studies, for example, to analyze the interactions between different types of ncRNAs involved in cardiac regulation and dysfunction. Indeed, researchers have used IntaRNA to predict potential interactions between long non-coding RNAs and miRNAs in which the first sponge out the second to achieve its downregulation in sepsis [184] and breast cancer [185].

Another example of such an approach is miRComb [164]. This R package (http://mircomb.sourceforge.net) infers miRNA–mRNA interactions by considering RNA hybridization from sequencing data but also by using databases as complementary information about putative interactions (seed complementarity, miRNA–mRNA complex statilities, and inter-species site conservation). This allows researchers to focus on the miRNA–target pairs most probable to be functionally relevant for the experiment under analysis. This tool was used to detect miRNA–mRNA interactions in ventricular myocardial biopsies from patients with HFpEF that were amenable to in vitro validation [117].

As stated before, different prediction algorithms frequently give rise to little overlap between them in terms of results. To overcome this hurdle, researchers frequently combine different target prediction databases as a way to find consensual information and/or predicted as well as experimentally validated targets [159]. Optimal miRNA targets thus obtained still need to be subjected to experimental tests by gene expression and functional experiments for final validation.

### 8.3. Bioinformatic Tools for Functional Analysis

Many studies use protein–protein interaction (PPI) network analysis to find target highly connected representative nodules of the miRNAs of interest. This can be conducted, for example, with data retrieved from the STRING database [165] or from the Human Integrated Protein–Protein Interaction rEference (HiPPIE) interactome database [166] and visualized using Cytoscape [30]. Indeed, STRING itself provides options for exporting data in formats compatible with Cytoscape to ease this workflow, such as SIF (Simple Interaction Format) or XGMML (eXtensible Graph Markup and Modeling Language). Target genes can similarly be used for functional overrepresentation analysis, such as Gene Ontology for functional analyses or the Kyoto Encyclopedia of Genes and Genomes (KEGG) or REACTOME [167] for pathway enrichment analysis (e.g., [186]). Interestingly, there is a dedicated consortium to annotate cardiovascular genes (over 4000) and they are also focusing on the GO annotation of miRNAs (http://www.ebi.ac.uk/QuickGO/GProteinSet?id=BHF-UCL (accessed on 18 December 2023); http://www.ucl.ac.uk/functional-gene-annotation/cardiovascular (accessed on 18 December 2023)). Finally, regulatory relationships can also be searched between transcription factors, miRNAs, and mRNAs from genes identified in those PPI networks (e.g., [187]). For this, a web-based gene set analysis toolkit, such as WebGestalt [168], or the database Transcriptional Regulatory Relationships Unraveled by Sentence-based Text-mining (TTRUST, [169]) can be used.

Integrated bioinformatics tools for the functional analysis of miRNAs have recently emerged for the automatic integration and functional interpretation of user-generated datasets that can be explored following different workflows: miRNA–mRNA expression correlation, target prediction, and functional enrichment analysis. These include DIANA miRPath [170] or miRGator [171]. Another good example is CopraRNA (Comparative prediction algorithm for small RNA targets), which starts by predicting targets based on IntaRNA but includes miRNA sequences from at least two other organisms and offers an array of downstream analysis, such as functional enrichment analysis, interaction domain identification, and regulatory networks [172]. These types of tools typically require specific input file formats for miRNA–target interaction data and may support additional input formats for other relevant information. For example, they need a mandatory file containing predicted or experimentally validated miRNA–target interactions (usually as plain text files or Excel spreadsheets) and optional inputs for pathway enrichment analysis (e.g., GSEA or other acceptable pathway database formats) or gene expression data for the samples under study (e.g., Excel, CSV).

### 8.4. Bioinformatics Tools for Disease Association

More recently, bioinformatics tools for ncRNA research have focused on highlighting deregulation and cataloguing unbalanced expression profiles of ncRNAs in human diseases. A very good example is miR2Disease, a manually curated database with information on miRNA-related pathologies [173]. miR2Disease curates several hundreds of miRNA–target interactions in humans, coupled with associated disease information derived from the literature consisting of more than 3000 miRNA–disease-related entries [26]. It is also possible to find information about miRNA expression and experimentally validated targets. Another example of a disease-related miRNA database is the Human microRNA Disease Database (HMDD). HMDD collects experimentally supported human miRNA–disease association data, including genetics, epigenetics, circulating miRNAs, and miRNA–target interaction [174]. Both databases are many times the starting point to creating disease-associated sets of deregulated ncRNAs that can be further studied in the context of a disease of interest, such as, for example, glaucoma [188].

Nevertheless, there are many more examples that can be highlighted in this group of tools, such as multiMiR, MISIM, or MIMRDA. multiMiR is both an R package and a database that integrates mRNA–target interactions for multiple databases together with disease and drug associations to identify the targets of miRNAs [175]. This tool often accepts plain text files or Excel spreadsheets containing lists of miRNAs or target genes for which the user wants to retrieve interactions and has been used, for example, in the context of bicuspid aortic valves [189]. MISIM is a web server to predict miRNA–disease associations based on positive or negative scores and provides network visualization and functional enrichment analysis for functionally paired miRNAs [176]. This tool is often used to simulate miRNA expression data in different biological conditions, and for that, it may require specific input files to specify conditions, miRNA profiles, or target gene information (often in plain text, CSV, or other structured formats). MIMRDA is a novel method to find top-ranked key miRNAs by incorporating miRNA and mRNA expression profiles for predicting miRNA associations with disease [177]. These approaches are normally carried out using generalist packages such as Limma [190] or SPIA [191], but MIMRDA seems to be superior for ncRNA research purposes [177]. MIMRDA is written in R; similar tools based on Python (e.g., MDPBMP [192] or miRModuleNet [193]) or MatLab (e.g., LGDLDA [194]) are available as well. For a complete review of this category of tools, please refer to [195].

### 8.5. Bioinformatics Tools for Integrative Data Analysis

miRToolsGallery is a bioinformatics resources database portal dedicated to small non-coding RNAs [178]. It allows researchers to search, filter, and rank the tools available within its database so that researchers of the field can find the right tools or data source for their studies. Among the available resources, researchers can also find review papers on the field. Unfortunately, the portal was last updated in 2018, but it gathers information for more than 1000 tools. Other databases of this kind include Tools4miRs (last update 2017 [160]), NRDR /NR2 (2018, [196]), and miRandb (2019, [179]).

miRNA Data Integration Portal (miRDIP) is a learning algorithm that is able to learn how to combine the score returned by several prediction algorithms as a way to discover miRNA regulatory networks from large-scale predictions [179]. It reconstructs all the possible multiple interactions between miRNAs and regulated gene networks. As an example, [197] used miRDIP together with the TargetScan and DIANA tools to predict miRNA targeting and to find which of the selected miRNAs could target co-DEGs in atrial fibrillation-related stroke. miRDIP typically allows users to submit lists of miRNAs and/or target genes of interest (commonly as plain text files, CSV files, or Excel spreadsheets). Likewise, this tool will return a list containing information about the miRNA interactions or associations based on the submitted input. These output files will present details, such as miRNA and/or target gene identifiers, and information about the strength or type of interaction. Optionally, miRDIP can generate graphical representations of miRNA–target interactions in standard image formats (PNG, SVG, or PDF).

Integrative omics data analysis platforms such as Galaxy [180], GeneMANIA [181], OMICtools [182], and others have been used to analyze multi-omics data, including ncRNA expression profiles, to identify novel regulatory networks and pathways, including those associated with heart failure.

In summary, the integration of various bioinformatics tools allows researchers in the cardiovascular field to comprehensively study the role of ncRNAs in cardiac pathology. These tools facilitate the annotation of novel ncRNAs, the identification of dysregulated ncRNAs, prediction of their targets and interactions, exploration of structural motifs, and elucidation of their involvement in signaling pathways, offering crucial insights into the molecular mechanisms underlying heart failure. However, researchers should consider the specific strengths and limitations of each tool while selecting the most suitable ones for their analyses. On the other hand, as stated before in the context of some of the tools presented here, the validation of results is a crucial step within bioinformatics since it ensures the reliability and accuracy of computational predictions or experimental findings. As in many other fields, the validation methods used in miRNA research can be broadly categorized into two main groups: computational validation (e.g., cross-validation, ROC or AUC-ROC analyses, etc.) and experimental validation (e.g., RT-PCR or microarrays for expression levels, reporter assays for miRNA–target interactions, etc.). Additionally, as the field of ncRNA research continues to evolve, new tools and updates to existing ones may provide enhanced functionalities and accuracy in ncRNA analysis.

## 9. Conclusions and Perspectives

Cardiovascular diseases constitute the leading cause of death worldwide according to the World Health Organization. Cardiac organogenesis is a complex developmental process that requires the orchestrated contribution of diverse cell types, which is regulated by intricate gene regulatory networks. Impairment in any of these developmental processes and/or gene regulatory networks invariably leads to cardiac congenital heart diseases, the most common type of congenital diseases in humans. In addition, adult cardiovascular diseases encompass a large array of cardiac abnormalities, including electrical, structural, and ischemic diseases that substantially contribute to mortality and morbidity in the human population. Understanding the molecular signaling pathways that contribute to cardiac homeostasis and disease is essential to provide adequate strategies to heal the damaged heart. In this context, increasing evidence has been reported on the pivotal role of microRNAs in cardiac development, congenital heart diseases, atrial fibrillation, heart failure, coronary artery disease, and myocardial infarction, as reviewed in this study. It is important to highlight in this context that microRNAs are also emerging as plausible sensitive biomarkers in different cardiac pathological conditions. For example, in the context of pediatric cardiovascular diseases, the discovery of placental expressed microRNAs in maternal plasma has been associated with congenital heart diseases [198]. Thus, they might be useful molecular markers for monitoring for pregnancy-associated cardiovascular diseases, opening new possibilities for early, non-invasive prenatal diagnosis and thus prevention, as recently reviewed by Omran et al. [199]. The identification of microRNAs as suitable biomarkers has also been extensively reported in major adverse cardiovascular events in atrial fibrillation [200], heart failure [201] and arrhythmogenic cardiomyopathies [202], among other cardiovascular diseases. Importantly, their use in prevention, treatment, and management is progressively emerging, although some additional efforts are required before they fully enter the clinical arena, such as, for example, providing adequate standardization for the methodology of purifying and detecting microRNA [200]. Nonetheless, the path to use microRNAs for the treatment of cardiovascular diseases is promising as clinical trials in other disease contexts have also been successful [203] and the first approaches are already in the way for heart failure [204].

More importantly, the recent discovery that microRNAs are selectively and distinctly loaded in extracellular vesicles (EVs) in different cardiac diseases has underpinned novel molecular mechanisms involved in cardiac homeostasis and disease. The identification of such novel intercellular communication pathways, mediated by extracellular vesicle (EV) trafficking, including their ample and variable microRNA cargo load, has opened new therapeutic approaches to heal the damaged heart. Thus, in coming years, additional applications of endogenous or experimentally modified EV delivery with selective microRNA cargos will become available for a larger number of cardiovascular diseases.

Similarly, current research on the molecular mechanisms that drive cardiac injury and regeneration has provided ample evidence that non-coding RNAs also play essential roles in these biological processes. In this context, it is important to highlight the continuous integration of bioinformatics-dedicated tools that could open up several new research directions using non-coding RNA-related therapies. Advanced data analytics and machine learning algorithms can be used to analyze complex biological data by combining multi-omics data, distinguishing patterns, and helping identify disease-specific miRNA signatures and their potential as therapeutic targets or diagnostic markers. Moreover, bioinformatics tools can be used for personalized therapies, analyzing individual clinical outcomes, and tailoring miRNA-based therapies accordingly to improve the efficacy of treatments and reduce potential side effects. Over the next coming years, we will witness further elucidation of the functional roles of these non-coding RNAs in cardiac injury and regeneration, thus paving the path to design novel strategies to heal the broken heart.

## Figures and Tables

**Table 1:** Bioinformatics tools traditionally used in ncRNA research.

Type/Goal	Tool	Reference
Discovery	miRDeep	[12]
	miRDeep2	[13]
	MiRFinder	[14]
	miReader	[15]
	RNAz2	[16]
Database and annotation	miRbase	[17]
	miRNet	[18]
	RNAcentral	[19]
Target prediction	miRanda	[20]
	TargetScan	[21]
	PicTar	[22]
	PITA	[23]
	miRDB	[24]
	RNAhybrid	[25]
Target databases	DIANA-TarBase	[26]
	MiRTarBase	[27]
	MiRWalk	[28]
Pathway analysis	DIANA Tools	[29]
	Cytoscape	[30]
Structure prediction	ViennaRNA Package	[31]
	MiRScan	[32]
	miRseeker	[33]

**Table 2:** miRNA in Heart Failure.

miRNA	Result	Reference
miR-18a	Targets *CTGF* and *TSP-1*	[118]
miR-19a	Targets *CTGF* and *TSP-1*	[118]
miR-21	Upregulated in HFpEF. Involved in cellular processes such as the proliferation, hypertrophy, apoptosis, and fibrosis of different cell types of the heart	[112]
miR-22	Targets *OGN*	[119]
miR-25	Upregulated in HFpEF. Inhibits *HAPLN1*mRNA	[117]
miR-26a	Upregulated in HFpEF. Inhibits *NPPB* and *HAPLN1* mRNA	[117]
miR-29	Targets extracellular matrix-related genes	[120]
miR-29a	Correlated with myocardial fibrosis	[121]
miR-30	Targets *CTGF*	[122]
miR-33a/b	Targets *PCK1* and *G6PC*	[123]
miR-103	Targets *CAV1* and *FASN*	[124]
miR-107	Targets *CAV1* and *FASN*	[125]
miR-125a	Upregulated in HFrEF. Induces M2 polarization in macrophages	[126]
miR-126	Targets *SPRED-1*	[127]
miR-133	Targets *GLUT4*	[128]
miR-138	Targets *S100A1*	[129]
miR-140	Upregulated in HFpEF. Inhibits *NPPB* mRNA	[117]
miR-146a	Targets *NFKB1*	[130]
miR-155	Targets *SOCS1*	[131]
miR-181a/c	Targets *IDH1*	[132]
miR-199a	Targets *GSK3B*	[133]
miR-210	Targets *ISCU*	[134]
miR-212	Targets *CACT*	[135]
miR-223	Targets *GLUT4*	[128]
miR-351	Targets *E2F3*	[136]
miR-370	Targets miR-122 expression	[137]
miR-378	Targets *PPARGC1B*	[138]
miR-4429a	Upregulated in HFpEF. Inhibits *HAPLN1* mRNA	[117]

**Table 3:** Examples of novel bioinformatics tools for ncRNA research.

Functionality	Tool	Possible Applications	Reference
**Family identification:**			
	Rfam	RNA annotation	[161]
	Infernal	Find homologous RNAs	[162]
**RNA–RNA interactions:**			
Prediction:	IntaRNA	Target prediction	[163]
	miRComb	Target prediction	[164]
**Functional analysis:**			
	STRING	Target expression retrieval	[165]
Based on expression	HiPPIE	Target interactome data retrieval	[166]
	Cytoscape	Network visualization	[30]
	Gene Ontology	Enrichment analysis	
Based on target function	KEGG	Enrichment analysis	
	REACTOME	Pathway enrichment analysis	[167]
Based on transcription regulation	WebGestalt	Find associated transcription factors, miRNAs, and mRNAs	[168]
	TTRUST	Find associated transcription factors, miRNAs, and mRNAs	[169]
Integrated tools	DIANA miRPath	Automatic functional interpretation based on the existing evidence (all of the above)	[170]
	miRGator		[171]
	CopraRNA		[172]
**Disease association:**			
	miR2Disease	Linking miRNA to disease	[173]
	HMDD	Retrieve miRNA–disease-associated omics data	[174]
	multiMiR	Use of disease and drug associations to find targets	[175]
	MISIM	Predict miRNA–disease association	[176]
	MIMRDA	Find top-ranked key miRNAs associated with diseases	[177]
**Integrative data analysis:**			
ncRNA-specific	miRToolsGallery (and similar)	Search, filter, and rank ncRNA-dedicated tools	[178]
	miRDIP	Find miRNA regulatory networks using ML methodologies	[179]
Generalistic	Galaxy	Build, run, and share bioinformatics pipelines using standard-use tools	[180]
	GeneMANIA	Explore functional relationships between miRNA and protein-coding genes	[181]
	OMICtools	Integrate multiomics data to find novel regulatory networks and pathways	[182]

Note: this is a non-systematic selection of frequently used tools, and many others can be used for the same purposes.

## Data Availability

Not applicable.
